# Shortcomings in the Current Amyotrophic Lateral Sclerosis Trials and Potential Solutions for Improvement

**DOI:** 10.3389/fneur.2017.00521

**Published:** 2017-09-29

**Authors:** Nakul Katyal, Raghav Govindarajan

**Affiliations:** ^1^Neurology, University of Missouri School of Medicine, University of Missouri, Columbia, MO, United States

**Keywords:** amyotrophic lateral sclerosis, clinical trials, animal models, biomarkers, genetic complexity, infrastructural issues

## Abstract

Amyotrophic lateral sclerosis (ALS) is a clinically progressive neurodegenerative syndrome predominantly affecting motor neurons and their associated tracts. Riluzole and edaravone are the only FDA certified drugs for treating ALS. Over the past two decades, almost all clinical trials aiming to develop a successful therapeutic strategy for this disease have failed. Genetic complexity, inadequate animal models, poor clinical trial design, lack of sensitive biomarkers, and diagnostic delays are some of the potential reasons limiting any significant development in ALS clinical trials. In this review, we have outlined the possible reasons for failure of ALS clinical trials, addressed the factors limiting timely diagnosis, and suggested possible solutions for future considerations for each of the shortcomings.

## Introduction

Amyotrophic lateral sclerosis (ALS) is a relentlessly progressive disease-causing widespread neuronal loss (1, 2). The disease follows a characteristic pattern, causing destruction of both upper and lower motor neurons (LMNs) (1, 2). However, the clinical spectrum can range from predominant upper motor neuron (UMN) to predominant LMN lesions (1, 2). The disease progression is persistent and eventually leads to death (1–4). Despite many previous and ongoing multimillion dollar research studies, a cure remains distant. There are many shortcomings in the past clinical trials that need to be addressed. In this review, we have attempted to address the potential reasons for the limited success in past ALS trials and have concluded that genetic complexity, inadequate animal study models, issues with trial design, insensitive biomarkers, and diagnostic delays are the main culprits hindering major development in ALS treatment. We have tried to address the challenges that complicate the search for a cure, while aiming to provide a preliminary understanding that may be helpful in formulating future studies.

## Genetic Complexity

### Background

About 90–95% of all reported ALS cases are sporadic (SALS), without any known family history and identifiable risk factors, whereas familial (FALS) accounts for 5–10% of all cases (1). An understanding of genes involved in ALS can be helpful in future for developing ideal therapeutic drugs.

Gene mutations associated with ALS are presented in Table 1.

**Table 1:** Genetic mutations associated with ALS.

Gene	Inheritance	Features	Underlying defect	Reference
SOD1	AD and AR	More than 20% of FALS, 1–2% of SALS	Superoxide metabolism	(4–6)
C90RF72	AD	More than 40% of FALS, 7% of SALS	DENN protein	(4–6)
TARDBP	AD	3% of FALS cases, 1% of SALS	RNA metabolism	(4–6)
FUS	AD and AR	5% of FALS, <1% of SALS	RNA metabolism	(4–6)

*(ALS,: amyotrophic lateral sclerosis; FALS,: familial amyotrophic lateral sclerosis; SALS,: sporadic amyotrophic lateral sclerosis; AD,: autosomal dominant; AR,: autosomal recessive; SOD,: superoxide dismutase; C90RF72,: chromosome 9 open reading frame 72; TARDBP,: TAR DNA-binding protein; FUS,: fused in sarcoma; DENN,: differentially expressed in normal and neoplasia; RNA,: ribonucleic acid.)*.

### Problems Related to Complex Genetic Association

A significant number of patients with apparent SALS carry an ALS-causing gene variant found in FALS patients (4). Among major mutations, C9ORF72 expansions are found in 7% of SALS patients, SOD1 in 1–2%, TARDBP in 1%, and FUS in <1% (4). This suggests that FALS-associated gene variants are present in up to 10% of patients with apparent SALS (4). Accordingly, it is increasingly recognized that because the characteristics of sporadic and familial disease overlap, differentiating between the two types is at times, challenging (4).

Given the predominant sporadic occurrence and rarity of the disease, gene mapping and identification of causative genes can be challenging (4). Genetic and phenotypic overlapping of SALS and FALS often results in misclassification of the two disease types (4). Patients with variable clinical subtypes are often pooled in the same study group, which later leads to significant statistical differences in the observations (4). Ascertainment bias can occur in patients with small family sizes, which can potentially cause misclassification of FALS as SALS (7). This is a commonly reported problem in patients with low penetrance familial disease variants such as the SOD1 mutations (7). As the penetrance of disease variant decreases, they are detected less often and more commonly misclassified as SALS (7, 8).

### Potential Solutions for Future Considerations

Amyotrophic lateral sclerosis can have multitudinous presentations, ranging from predominant UMN or LMN features to non-motor symptoms; this heterogeneity poses significant diagnostic challenges (4). Understanding the genetic constituent of each disease-causing variant can help in delineating the underlying pathophysiology (4). There is a great need to extensively study all the genetic models and tailor a focused clinical study on each individual model (4). Studies should be formulated taking genotypic features into consideration to avoid discrepancies in recognizing the ALS subtypes (4). Understanding individual models and formulating focused clinical trials can be the cornerstone in the development of effective therapeutic agents (4).

## Inadequate Animal Models

### Background

Multiple disease-causing mutations and effect of therapeutic drugs on disease progression have been tested in animal models (9–11). These animal models include mice, rat, and canine models.

### Mice Models

Mice models were the first studied ALS animal models (9, 10). SOD1^G93A^ coupled with human SOD1 promoter was tested in mice models, which replicated most of the ALS characteristics seen in humans (10).

Frey et al. studied three genetic mouse models with motor neuron disease of different origin and severity (10, 11). In their study, motor dysfunctions were seen at around 80–90 days after neuronal dysfunction and death occurred at around 130 days (10, 11). Studies showed that neuronal destruction preceded the clinical onset and was evident at around 40–50 days (12). Previously, it was believed that loss of dismutase function was the underlying reason for the observed clinical effects; however, it was seen that instead of motor neuron loss, SOD1 mice models developed distal motor axonopathy resulting in motor dysfunctions (13, 14). SOD1 mice models noticeably had mitochondrial vacuolization, but neuronal destruction was not evident until 2 years (15–17). Another study reported predilection for development of bladder symptoms in SOD1^D90A^ mice models (10, 18) (Table 2).

**Table 2:** Characteristics of genetic mutations tested in various animal models.

Animal model	Mutation	Age at onset (weeks)	Survival (weeks)	Neuropathological findings	Reference
Mouse	G93A	13–17	17–26	MN loss, SOD1 aggregates, NMJ loss before onset	(9–18, 48–51)
	G37R	15–17	25–29	Learning deficit MBV, LMN first affected, raised somatosensory thresholds	(9–18, 48–51)
	D83G	15	70–84	Sensory deficit, tremors, 20% LMN and UMN loss	(9–18, 48–51)
	D90A	52	61	Distended bladder, SOD1 inclusions, MN loss	(9–18, 48–51)

Rat	H46R	20	24	MN loss, LBHI, SOD1–ubiquitin aggregate	(10, 19, 20)
	G93A	16	17	MN loss, vacuoles, SOD1–ubiquitin inclusions	(10, 19, 20)

Dog	T18S	7 years	21 months	SOD1 aggregates no neuronal cell body loss, UMN and LMN signs, sensory impairment	(10, 52, 53)
	E40K	>5 years	6 months to 3 years		

*SOD1, superoxide dismutase 1; MN, motor neuron; NMJ, neuromuscular junction; MBV, membrane bound vesicles; UMN, upper motor neuron; LMN, lower motor neuron; LBHI, Lewy body-like hyaline inclusion*.

### Rat Models

H46R and G93A variants of SOD1 have been studied in rat models (10). These variants also reportedly produced UMN and LMN degeneration (10). SOD1^G93A^ models showed findings corresponding to bulbar ALS with loss of motor neurons in the trigeminal, facial, and hypoglossal nuclei (10, 19). The majority of the randomized controlled trials (RCTs) for disease-modifying treatments have been tested on rat models (20). Drugs tested in numerous RCTs over the year are described in Table 3 (21–47).

**Table 3:** List of drugs used in randomized control trials for potential treatment of Amyotrophic lateral sclerosis, their possible mechanism of action, and reasons for trial failure.

Drugs tested	Possible mechanism	Reason for failure	Reference
Transfer factors	Antivirals	Weak rationale	
Riluzole, threonine, lamotrigine, gabapentin, topiramate, memantine	NMDA receptor blocker, GABA-analog, and glutamate AMPA receptor blocker antagonists, increases astrocytic glutamate transporter activity	NMDA receptors are not critical for motor neurons	(21–47)
Octacosanol, gangliosides, thyrotropin-releasing hormone, growth hormone	Myotrophic effects, systemic trophic factors	Weak rationale	(21–47)
CNFT, IGF-1, BDNF, GDNF, Xaliproden, GCSF	Retrograde transport from the muscle axon terminals, serotonin (5HT1A) agonist	Drugs unable to cross the blood–brain barrier	(21–47)
Plasma exchange, cyclosporine, total lymphoid irradiation, glatiramer acetate	Humoral factors, T-cell, microglial suppressor	Weak rationale	(21–47)
Acetylcysteine, glutathione, vitamin E	Increases antioxidative property	Uncertain access to the nervous system	(21–47)
Pentoxifylline, minocycline	TNFα-linked apoptosis	Weak rationale	(21–47)
Creatine, acetyl l carnitine	Mitochondrial membrane stabilizing drugs	Weak rationale	(21–47)
Phenylbutyrate, valproic acid	Histone deacetylase inhibitor	More studies are ongoing	(21–47)
Lithium carbonate, pioglitazone	Degradation of protein aggregates	Weak rationale	(21–47)
ONO 2506	Blocks gliosis	Negative studies	(21–47)

*NMDA, N-methyl d-aspartate; GABA, gamma aminobutyric acid; CNFT, ciliary neurotrophic factor; IGF, insulin-like growth factor; GCSF, granulocyte colony-stimulating factor; BDNF, brain-derived neurotrophic factor; GDNF, glial cell line-derived neurotrophic factor; ONO 2506, enantiomeric homolog of valproic acid developed by ONO pharmaceuticals*.

### Canine Models

Studies have shown that a neurodegenerative disorder in dogs presents with similar gene mutations as seen in ALS (52, 53). According to genome-wide association studies (52), dogs have lesser well-shuffled genomes than humans, making them somewhat ideal study models for genetic studies. T18S and E40K mutations affecting the SOD1 gene reportedly share similarities with canine degenerative myelopathy, a fatal neurodegenerative disease affecting dogs (10, 52, 53). Thus, canine study models may pave the way for future successes in finding ideal therapeutic targets for ALS.

Table 2 presents characteristics of genetic mutations tested in various animal models.

### Challenges with Animal Models

Animal model studies have numerous methodological flaws, in particular, treatment is usually started before onset of symptoms in the majority of RCTs (47). SOD1 mice models were treated with therapeutic agents before the disease onset, which likely provided neuroprotection (47). Furthermore, the neuroprotective effects of therapeutic drugs and survival duration were overestimated in clinical trials conducted in mice models (47). The ALS Therapy Development Institute (TDI) trial, which tested around 100 potential therapeutic drugs in mice models, has confirmed the overestimated survival duration reported in the majority of previous therapeutic clinical trials (54). The trial proposed possible reasons for overestimated survival, which included failure to exclude mice deaths from causes other than the disease under study (54, 55). Gender-specific ALS presentation was not taken into consideration in multiple RCTs (54, 55). Mice SOD1 models showed earlier onset of symptoms and died week before female mice (54, 55). Such variability in survival could have been mistaken as a potential drug effect (54, 55). Like in many other diseases, ALS, animal models carry multiple copies of the disease-causing gene; however, all genes may not pass to subsequent generations, thereby resulting in loss of disease phenotype (54, 55).

### Potential Solutions for Future Considerations

The ALS-TDI trials recently introduced the latest guidelines to limit unwanted clinical trials with misleading conclusions, which may be, helpful in formulating future studies (54). The guidelines include:

- Performing rigorous assessment of physical and biochemical traits of animal models and characterizing when disease symptoms and deaths occur and being alert to unexpected variation (54).
- To reduce false conclusions, it is suggested that male and female mice be separate in different groups, as they can show variations in symptom development and survival (54).
- Symptoms should be periodically reported to study variations in the occurrence pattern (54).
- Gene tracking is also highly recommended, as not all disease-causing genes are passed onto subsequent generations (54).
- Randomization and blinding should be implemented in animal studies to limit spurious conclusions (54).

## Issues with Trial Design

### Background

More than 50 RCTs have been conducted in the past few decades to develop an effective therapeutic target for ALS (56). FDA has only approved 16% of all therapeutic interventions till date, used in trials by pharmaceutical companies (56). Such statistics clearly indicates the critical need for thorough reassessment of the current conducting methods being used in therapeutic development. Multiple therapeutic agents have been studied in past RCTs, including antioxidants, antiapoptotic agents, and neurotrophic factors (57).

Table 3 presents a list of the various drugs tested in past RCTs, their possible mechanism of action, and the reason for trial failure (21–47).

### Problem with Trials

While more than 18 different drugs have been tested in phase 2 or 3 RCTs, none of them have emerged as an effective therapeutic agent (21–47).

Mitsumoto et al. (47) proposed potential reasons for negative results from RCTs and classified them into three broad categories: inappropriate trial rationale, pharmacological issues, and clinical trial design issues.

With respect to the trial’s rationale, studies reported that almost two-thirds of negative studies were due to apparent misleading positive results reported in SOD1 mice (47). Fourteen (78%) of 18 RCTs were based on previously positive SOD1 preclinical studies (47). SOD1 models failed to recapitulate similar results when tested in humans (20, 47). The majority of RCTs were reported to have significant pharmacological issues, including doses being too low, *U*-shaped effectiveness curves, problem with CNS access, and absence of pharmacokinetic and pharmacodynamic analyses (47). However, the most commonly reported problem was of potential drug interaction (47). The majority of RCTs testing new drugs were conducted on patients who were previously on riluzole therapy (21–47). Pharmacological interactions of drugs under study and effects of riluzole were not taken into consideration during RCTs (47).

The other reported concern was regarding clinical trial design and methodological issues. Investigators raised questions about the variability in disease presentation, enrollment of patients with advanced disease, and short study duration (47).

Every year, a large number of studies are conducted with the hope of developing effective therapeutic solutions for ALS, but only few reach conclusion and get published (47). Lack of publications of many negative clinical trials lead to repetition of the same study rationale elsewhere, thereby wasting valuable time and resources (47).

### Potential Solutions for Future Considerations

In order to optimize results, trial designs should be modified according to the population under study, route of drug delivery, and phase of clinical trials (58).

#### Population under Study

Phenotypic heterogeneity of ALS poses significant challenges in classifying the study population (4). The majority of studies enroll patients regardless of their clinical subtype in the same study group, which confers statistical challenges (4). Recent trends suggest that studies should be formulated taking genotypic features into consideration to avoid discrepancies in recognizing the ALS subtypes (4, 58).

#### Route of Delivery

Although intrathecal or intramedullary delivery can be difficult to achieve, it can effectively address concerns regarding inadequate CNS drug dosage (58). Current study designs are being formulated to optimize intrathecal and intramedullary drug delivery so as to effectively bypass the blood–brain barrier and maximize the drug effect (58).

#### Modification of Traditional Study Design

Clinical trials can be divided into Learning Phase and Confirmatory Phase (58). The learning phase incorporates studies on learning potential toxicity, drug interaction, and the pharmacokinetic and pharmacodynamic parameters of a therapeutic agent (58). The confirmatory phase focuses on standard phase III randomized, placebo-controlled trial design, and aims to determine drug efficacy and safety (58). The combination of learning and confirmatory adaptive designs may further promote ALS trial efficiency and statistical power.

## Insensitive Biomarkers

### Background

Biomarkers are objectively measured agents that indicate either a normal biological process, or a pathological process, or a biological response to a therapeutic intervention (58, 59). A reliable progression marker would make it possible to conduct shorter trials, on a smaller number of patients, thereby opening up the prospect of more diversified trials (60, 61). Despite intensive research spanning the past 20 years, there are currently no practical diagnostic biomarkers for ALS (58–61). This often leads to diagnostic delays before the appropriate treatment is administered (58–61). Neurophysiological approaches, such as electromyography and motor unit number estimation (MUNE), play a key part in detecting LMN pathology (62, 63). However, these methods do not always reliably monitor disease progression and treatment effects (62–66). While advanced techniques, such as motor unit number index (MUNIX), Bayesian MUNE, and electrical impedance myography, are more accurate, they still need further validation against neuropathological correlates (64–66).

### Source of Biomarkers

Cerebrospinal fluid is an important source of biomarkers, as it communicates directly with the brain parenchyma. Hence, it contains proteins and metabolites, at a relatively higher concentration than in other fluids and can indicate the presence and extent of a neurodegenerative process (58). Blood appears to be the most suitable source for biomarker discovery, as it is easy to access and handle and allows minimally invasive multiple testing at a low cost (61, 67). On the other hand, it must be assumed that its composition is affected by biochemical changes in the brain and the spinal cord as a result of a pathological process (61, 67).

The FDA recognizes four different types of biomarkers based on their utility in drug-development trials (58, 68):

1. Diagnostic biomarkers possess characteristics that can categorize patients by the presence or absence of a specific physiological or pathophysiological state or disease (58, 68).
2. Prognostic biomarkers possess characteristics that can categorize patients by the degree of risk for disease occurrence or progression of a specific aspect of disease (58, 68).
3. Predictive biomarkers possess baseline characteristics that can categorize patients by their likelihood of response to a particular treatment relative to no treatment (58, 68).
4. Pharmacodynamic biomarkers possess characteristics that can show that a biological response has occurred in a patient who has received a therapeutic intervention (58, 68) (Table 4).

**Table 4:** Biomarkers associated with amyotrophic lateral sclerosis and their potential benefits.

Biomarkers	Potential benefits	Reference
CSF pNfH	Prognostic biomarker	(69–72)
CSF NfL	Prognostic biomarker	
Urinary p75	Prognostic biomarker, shows progression and has pharmacodynamic property	(69–72)
CSF SOD1	Pharmacodynamic property	(69–72)
CMAP	Shows progression	(69–72)
MUNE	Shows progression	(69–72)
MUNIX	Shows progression and has pharmacodynamic property	(69–72)
EIM	Shows progression and has pharmacodynamic property	(69–72)
Peripheral Nerve Excitability Testing	Shows progression	(69–72)
TMS	Shows progression	(69–72)
SOD1 gene mutation	Potential predictive properties	(69–72)
Hexanucleotide repeat expansion in the C9orf72		

*CSF, cerebrospinal fluid; NfH, phosphorylated neurofilament heavy chain; NfL, phosphorylated neurofilament light chain; SOD, superoxide dismutase; CMAP, compound motor action potential; Urinary P 75, urinary p75 neurotrophin domain; MUNE, motor unit number estimation; MUNIX, motor unit number index; EIM, electrical impedance myography; C90RF72, chromosome 9 open reading frame 72; SOD1, superoxide dismutase 1; TMS, transcranial magnetic stimulation*.

An ideal prognostic biomarker should change in response to disease progression as well as to the introduction of a therapeutic intervention (69). The most promising biomarkers for ALS therapy development described till date can be broadly classified into biological fluid-based biomarkers and electrophysiological biomarkers (69).

### Biological Fluid-Based Biomarkers

1. Phosphorylated neurofilament heavy and light chains: the neurofilament subunit proteins NfH and NfL, are found in blood and CSF in multiple pathological processes, including ALS (69). They can be easily detected by conventional antibody-based immunoassays (69). Higher levels of NfL correlate with faster future disease progression and shorter survival (69, 73)
2. p75 neurotrophin domain: neurotrophin p75 (p75NTR) stimulates neuronal cells to differentiate (69). Injury to nerves and schwann cells can lead to shedding of p75NTR from cell membranes, facilitated by neurotrophin action (69). A study showed that p75NTR is excreted into the urine of SOD1 mice and humans with ALS (69). The study also described changes in the expression of SOD1 in tissue and biological fluids for total and misfolded SOD1 (69).

### Electrophysiological Markers

Electrophysiological markers such as MUNE; MUNIX, and compound motor action potential (CMAP) are extensively utilized not only to diagnose ALS, but also to monitor disease progression and effects of therapeutic interventions (69).

1. CMAP: reduced CMAP amplitude is one of the characteristic findings in ALS (69). Amplitude reduction correlates to the underlying axonal loss and is characteristically seen at the time of ALS diagnosis (69).
2. MUNE: MUNE, an electrophysiological biomarker, is commonly used for evaluation of ALS progression (69). It calculates the number of motor neurons innervating a particular muscle (69). Calculation of both MUNE and MUNIX is derived from CMAP (69).
3. Electric impedance myography (EIM): EIM measures conductive and capacitive properties of muscle groups by applying small high-frequency electric current (69, 74). EIM provides electro morphological data rather than being an electrophysiological marker. Multiple studies have proved its high reliability and sensitivity in monitoring disease progression (66, 69, 74–76).
4. Peripheral nerve excitability testing: excitability testing measures electrotonus threshold, strength duration time constant, and the recovery cycle (69, 77, 78). Studies have reported association of higher level of excitability with reduced survival (79).
5. Transcranial magnetic stimulation (TMS): multiple studies have reported the association of ALS with increased cortical excitation, an observation derived by TMS studies (69, 80–82). Other TMS measures such as motor threshold, motor evoked potential, cortical silent period, and central motor conduction time are also altered in ALS (69, 83).

### Problems with Biological Biomarkers

Interpretation of biological fluid-based biomarkers can be challenging, as errors are known to occur in both the pre-analytic and analytic phases (69, 84).

### Pre-Analytic Phase Issues

#### Study Designs

Ideally, study designs for evaluation of diagnostic biomarkers should differentiate ALS from other ALS mimics that can pose diagnostic challenges (69). Studies have reported that use of case-control design for diagnostic evaluation tends to overestimate the sensitivity and specificity of diagnostic tests (69, 84). Similarly, longitudinal studies have significant risk of loss of patient follow-up as the disease progresses (69).

#### Confounders

Age, ethnicity, gender, and comorbidities are all known to confound the association between biomarkers and clinically relevant phenotypic features (69).

#### Variabilities

Multiple factors can potentially introduce significant degree of variability that can influence biomarker quantification (69). Differences in sample collection, processing, storage, and diurnal fluctuations in biomarker levels can cause significant variation in measurement (69).

### Analytic Phase Issues

#### Problem with Electrophysiological Biomarkers

All electrophysiological biomarkers utilize parameters like CMAP, MUNIX, and MUNE that can only be obtained from nerves and muscles that are effectively stimulated (69). Electrophysiological biomarkers have their own challenges, the most important being repeatability, which can significantly vary with discrepancies in electrode positioning, limb and hand positioning, electrode size, and limb temperature (69). Compared with CMAP, MUNE and MUNIX are somewhat superior electrophysiological markers, as they can indicate disease progression at much earlier stages, when CMAP size is apparently normal, owing to ongoing reinnervation (69–72). However, MUNE can be challenging to perform and requires expertise (69–72). Furthermore, it is associated with high test–retest variation, which significantly limits its utility (69–72). MUNIX is relatively easier to obtain; however, its reliability has not been extensively evaluated (69–72).

Nonetheless, extensive research studies are required to ascertain the use of biological and electrophysiological biomarkers as an indicator of disease progression in ALS (69).

### Potential Solutions for Future Consideration

Potential biomarkers should also be evaluated in more appropriate control conditions that can mimic ALS, the exclusion of which causes regular diagnostic delays during early disease stages, when patients present with only UMN or LMN signs (85). Different studies on the same candidate biomarker can sometimes produce contradictory results (85). To avoid this inconsistency, both the choice of well-defined individuals and the standardization of quantification methods should be mandatory (69, 85). To address the analytical phase issues and clinical validation of the immunoassay studies, the FDA has recommended specific guidelines that pertain to documentation of the specificity of the immunoassay, sensitivity for detecting the specific biomarker in the biological fluid of interest, precision and accuracy of the method, and robustness over different days and in different laboratories (69, 86). Development of biomarker assays according to the FDA guidelines can provide uniformity in characterization of analytical performance of immunoassays (69, 85, 86).

An approach similar to earlier biomarker initiatives such as the Parkinson Progression Markers Initiative and the Parkinson Disease Biomarkers Program (69) is required to collaborate large pharmaceutical companies along with academic centers to form an ALS Biomarker Consortium in order to develop effective biomarkers (69). Northeast lateral amyotrophic lateral sclerosis consortium have undertaken a novel initiative and developed a repository of serum, plasma (CSF), and other biological fluid samples from patients with ALS and motor neuron diseases to implement research studies for therapeutic development and biomarker testing (69).

### Diagnostic Delays

Diagnosis of ALS is often delayed and is generally reported months after commencement of neuronal destruction (57). By the time patients enter a clinical trial, their disease may be too advanced for the drug to work (57). The potential reasons for delay in diagnosis include; the general perception of people to avoid visiting physicians for vague symptoms and wait until they are questionably ill (87). Moreover, earlier presentation of ALS symptoms can be confused with multiple other neurological processes including spinal cord diseases, mononeuropathies, and several neurological syndromes that further delay the diagnosis (87). Delayed referral to a neurologist is one of the major reasons for delayed diagnosis (87, 88). According to one observational study, after first consultation by a general practitioner, the referral to a neurologist, on average, takes 7 months (87–89). The average duration between presentation of first symptoms to diagnosis of ALS is 9.3 months, according to El Escorial and Airlie House criteria (87–90). Time to diagnosis can be further prolonged in patients with spinal onset of ALS and age between 65 and 75 years (87, 89). Limited trial centers for ALS also significantly hinder the access to quality health care and enrollment in trial studies (87, 88).

The El Escorial and Awaji diagnostic criteria utilizes clinical, electrophysiological, and imaging parameters and classify ALS based on diagnostic certainty (87). The accuracy can be limited by multiple variations in ALS presentation (87) (Table 5).

**Table 5:** Revised El Escorial criteria for diagnosis of amyotrophic lateral sclerosis (ALS).

Clinically definite ALS	Evidence of UMN plus LMN signs in the bulbar region and in at least two spinal regions or Presence of UMN signs in two spinal region and LMN signs in three spinal regions	(87)
Clinically probable ALS	Evidence of UMN plus LMN signs in at least two regions with some UMN signs rostral to LMN signs	(87)
Probable, laboratory-supported ALS	Clinical evidence of UMN and LMN signs in only one region or UMN signs alone in one region and LMN signs defined by EMG criteria in at least two muscles of different root and nerve origin in two limbs	(87)
Possible ALS	UMN and LMN signs in only one region, or UMN signs alone in two or more regions, or LMN signs found rostral to UMN signs	(87)

*Regions: bulbar, cervical, thoracic, lumbosacral*.

*UMN, upper motor neuron; LMN, lower motor neuron*.

### Potential Solutions for Future Considerations

There is a strong need for neurologists to undertake a collaborative effort to incorporate various patient organizations to raise public awareness for ALS (91). To ensure early referral to a neuromuscular specialist, general practitioners should be educated about the early symptoms of illness and the importance of electrophysiological criteria for accurate diagnosis (87, 91). To overcome the limitation of inadequate trial centers, small community-based centers should be registered to larger university trial centers, adopting universal enrollment and monitoring criteria for affected patients (87, 91). Telemedicine and other latest advances to target home-based care for providing improved access to patients should be implemented (57).

## Conclusion

Amyotrophic lateral sclerosis is a relentlessly progressive motor neuron disease with high mortality. Despite decades of extensive research and numerous RCTs, no effective therapeutic intervention or diagnostic biomarker has thus far been developed. In this review, we have attempted to provided potential solutions for major problems plaguing any significant development in ALS clinical trials, which can be helpful in formulating future studies. The most important goal for the next decade of ALS research should be a multidisciplinary approach collaborating with international ALS clinical trials, funding agencies, pharmaceutical companies, basic scientists, and patient advocacy groups to formulate an adaptive clinical study design to test drugs with possible biological and therapeutic targets in ALS (47).

### Search Strategy and Selection Criteria

We searched PubMed records between January 1, 1980, and January 1, 2016, and retrieved references from relevant articles. The search terms included “Amyotrophic lateral sclerosis,” “Motor Neuron Disease,” and “Randomized Control Trials,” every drug or therapeutic agent used in the review was used in the search list. There were no language restrictions. The final reference list was generated on the basis of relevance to the topics covered in the review.

## Author Contributions

NK and RG: literature review and review writing.

## Conflict of Interest Statement

The authors declare that the research was conducted in the absence of any commercial or financial relationships that could be construed as a potential conflict of interest. The reviewer VG and handling editor declared their shared affiliation.
